# Psychometric property study of the Affective Lability Scale-short form in Chinese patients with mood disorders

**DOI:** 10.3389/fpsyt.2023.1160791

**Published:** 2023-04-04

**Authors:** Mohan Ma, Chuman Xiao, Wenwen Ou, Guanyi Lv, Mei Huang, Xiaotian Zhao, Yaqi Qin, Yumeng Ju, Yan Zhang

**Affiliations:** ^1^Department of Psychiatry, Second Xiangya Hospital, Central South University, Changsha, Hunan, China; ^2^Mental Health Institute of Central South University, China National Clinical Research Center on Mental Disorders (Xiangya), China National Technology Institute on Mental Disorders, Changsha, Hunan, China

**Keywords:** Affective Lability Scale-short form, mood disorders, major depressive disorder, bipolar disorder, reliability, validity

## Abstract

**Introduction:**

This study aimed to investigate the psychometric properties of the Affective Lability Scale-short form (ALS-SF) among Chinese patients with mood disorders, and to compare ALS-SF subscale scores between patients with major depressive disorder (MDD) and patients with bipolar disorder (BD) depression.

**Methods:**

A total of 344 patients with mood disorders were included in our study. Participants were measured through a set of questionnaires including the Chinese version of ALS-SF, Patient Health Questionnaire-9 (PHQ-9), Generalized Anxiety Disorder 7-item (GAD-7), and NEO-Five Factor Inventory (NEO-FFI). Exploratory factor analysis and confirmatory factor analysis were applied to examine the psychometric properties of ALS-SF. Besides, correlation and regression analyses were performed to explore the relationship between affective lability and depression, anxiety, and neuroticism. Independent samples *t*-tests were used to compare the subscale scores of ALS-SF between the MDD and BD depression groups.

**Results:**

Results of factor analysis indicated that the model of ALS-SF was consistent with ALS-SF. The ALS-SF showed a solid validity and high internal consistency (Cronbach’s alpha = 0.861). In addition, each subscale of ALS-SF was significantly correlated with PHQ-9, GAD-7, and NEO-FFI neuroticism subscale, except for the anger subscale showed no significant correlation with PHQ-9. Besides, the depression/elation and anger factor scores in patients with BD depression were higher than in patients with MDD.

**Conclusion:**

Our study suggests that the Chinese version of ALS-SF has good reliability and validity for measuring affective lability in Chinese patients with mood disorders. Assessing affective lability would assist clinicians to distinguish between MDD and BP depression and may decrease the risks of misdiagnosis.

## 1. Introduction

Affective lability refers to the tendency for emotions to change rapidly, unpredictably, and excessively (1), which is one of the important features in mood disorders including major depressive disorder (MDD) and bipolar disorder (BD) (2–4). However, affective lability is not clearly defined or listed as a diagnostic criterion of MDD and BD in mainstream clinical diagnostic tools such as DSM-V or ICD-11, and only a small number of scales quantify affective lability regarding transitions between different emotions (i.e., depression, anxiety, anger, and mania). Considering that affective lability is closely related to the development of MDD and BD, a valid tool is needed to systematically assess affective lability in patients with MDD and BD.

Several studies have shown that affective lability is strongly related to the development and adverse outcomes of MDD and BD. A previous prospective study indicated that affective lability was a precursor of MDD (5). Affective lability was also found to be associated with depression *via* negative cognitive biases (6) including attention, memory, and the interpretation of ambiguous information (7, 8), and negative cognitive biases would further contribute to the recurrence of MDD (9). In terms of BD, affective lability was proved to present not only in the early stage (10), inter-episode, and depressive episodes of BD (11, 12) but also present in unaffected first-degree relatives (13, 14). A prospective cohort study found that affective lability would increase as youths with a family history of BD progress from asymptomatic to the onset of symptomatic BD (14). Given that ALS is significantly associated with disease progression and adverse clinical outcomes, assessing affective lability is essential in both scientific research and clinical practice, especially in treating patients with mood disorders.

One of the most used scales that measure affective lability is the Affective Lability Scale-54 (ALS-54) developed by Harvey et al. (15). The ALS-54 measures the tendency of rapid transitions from the different emotional states of anxiety, depression, anger, and elation, as well as changes between depression and elation, and depression and anxiety. Considering that the 54-item ALS could be too lengthy to be applied in clinical settings, an 18-item short version of the ALS (ALS-SF) based on a three-factor model (anxiety/depression, depression/elation, anger) was then developed (16) and found highly correlated with the original version (*r* = 0.94) (17). Psychometric properties of the ALS-SF have been validated and replicated in healthy samples (18), personality disorders (17), BD (19), and attention deficit hyperactivity (20) in various countries. Furthermore, while the Chinese version of ALS-SF has been validated in the general adolescent population (21), its reliability and validity in the Chinese adult population and in patients with mood disorders remain unknown. Therefore, the purpose of this study was to examine the psychometric properties of the ALS-SF for Chinese patients with mood disorders and to investigate the relationships between affective lability and depressive symptoms, anxiety symptoms, and neuroticism. Our study also aims to explore differences in affective lability between patients with MDD and with BD depression.

## 2. Methods

### 2.1. Participants and procedures

A total of 344 patients were recruited from the outpatient and inpatient departments of Second Xiangya Hospital of Central South University in Changsha, China. Participants include 221 patients with MDD and 123 patients with BD who were diagnosed by psychiatrists using Mini International Neuropsychiatric Interview for DSM-V (MINI 7.0.2 for DSM-5). Inclusion criteria for participants in this study were (1) 18–60 years of age; (2) signed informed consent to participate in this study and were able to complete the interview and scale; and (3) diagnosed with MDD or BD (types I, II or cyclothymic) with or without general anxiety disorder based on the DSM-V diagnostic criteria. Exclusion criteria were patients with (1) any history or current co-morbid psychiatric diagnoses that met the DSM-V criteria for any Axis I disorder other than MDD, BD, and general anxiety disorder; (2) existence of psychotic symptoms; (3) severe physical disorders such as rheumatic immune system diseases, endocrine, and metabolic diseases, nervous system diseases, etc.; and (4) intellectual disability. Prior to data collection, all participants were informed of the purpose and procedure of this study. Questionnaires that with obvious filling errors were excluded after data collection. The study protocol was approved by the Ethics Committee of the Second Xiangya Hospital of Central South University.

### 2.2. Measurements

Variables collected include demographic and clinical characteristics. Demographic characteristics consist of age (years), gender (male/female), educational level (years), family income (CNY), and residence (urban/rural). Clinical variables involve current illness duration (months), and total illness duration (months).

Affective lability was assessed using the Affective Lability Scale-short form (ASL-SF) (16), which was authorized by Professor Jeffrey S. Simons from the University of South Dakota. The Chinese version of ALS-SF was translated by two psychiatrists proficient in English. One psychiatrist translated the original version into Chinese, then another psychiatrist translated it back into English to ensure the adaptation was valid. The Chinese version used in this study was presented in the Supplementary material. This Chinese version of ALS-SF adapted to adolescents has once been validated in Chinese adolescents (21). The original scale consists of a three-factor model assessing the transitions between anxiety and depression (item 1, 3, 5, 6, 7), depression and elation (item 2, 10, 12, 13, 15, 16, 17, 18), and anger (item 4, 8, 9, 11, 14). Participants were asked to rate their emotional state in the past week on a 4-point Likert scale (0 = “very uncharacteristic of me” to 3 = “very characteristic of me”) for each item on the ALS-SF. A higher total score indicates a higher level of affective lability.

Depression and anxiety were measured by the Chinese version of the Patient Health Questionnaire-9 (PHQ-9) and the Generalized Anxiety Disorder (GAD-7), which showed good reliability and validity in patients with mental disorders and the general population (22–25). Each item in the PHQ-9 and GAD-7 is rated on a 4-point Likert scale ranging from 0 to 3 (0 = “not at all” to 3 = “nearly every day”). Total scores of the PHQ-9 and GAD-7 range from 0 to 27 and 0 to 21 respectively, with a higher score suggesting a higher level of depression or anxiety.

The NEO-Five Factor Inventory (NEO-FFI) is a 60-item, 5-domain comprehensive scale (26) assessing individuals’ five types of personality traits/subscales including extraversion, agreeableness, conscientiousness, neuroticism, and openness. Each item is rated on a 5-point Likert scale, ranging from 1 to 5 (1 = “strongly disagree,” 5 = “strongly agree”). Our study mainly focuses on the neuroticism trait (Neuroticism subscale) as affective lability is found to be closely related to neuroticism, which is often used to describe the degree of emotional instability or negative emotions (27).

### 2.3. Statistical analyses

Data analyses were performed in the Statistical Package for the Social Sciences (SPSS) 23.0 and Mplus 8.0. For demographic and clinical characteristics, continuous variables were presented as appropriate for median and interquartile range or mean and standard deviation. Frequency and percentages for each category were analyzed to present categorical variables. Cronbach’s coefficient alpha (α) was calculated to examine the internal consistency of the ALS-SF. The Cronbach’s α > 0.7 indicates a high internal consistency (28).

Exploratory factor analysis (EFA) and confirmatory factor analysis (CFA) were performed to further evaluate the reliability and validity of the ALS-SF. The whole dataset was randomly separated into two groups to run EFA and CFA. The value of Kaiser–Meyer–Olkin (KMO) and Bartlett’s test of sphericity was calculated to determine the appropriateness of performing factor analysis. The KMO > 0.7 and Bartlett’s test of Sphericity’s *p* < 0.05 indicate the dataset is appropriate for factor analysis. Maximal rotation of variance and principal axis factoring (PFA) were conducted to perform EFA to examine the validity and factor structure of ALS-SF (29). And then, CFA would be performed on factors whose eigenvalues are greater than 1 and items whose factor loadings are greater than 0.5 in the results of EFA to examine the model structure. Composite Reliability (CR) reflects the internal consistency of items within each factor, and it would be considered adequate when CR > 0.7. The goodness-of-fit of the factor model was assessed using a selection of fit indices and criteria: root-mean-square error of approval (RMSEA) < 0.08, standardized root-mean-square residual (SMSR) < 0.08, and comparative fit index (CFI) > 0.9 (30).

The convergent validity and divergent validity of the ALS-SF were assessed by factor loading and Average Variance Extracted (AVE). Factor loading measures the correlation between items and factors. AVE measures the level of variance of each factor explained by the construct of the 3-factor model compared to the level of variance due to measurement error. Convergent validity would be adequate if the scale items are significantly correlated with the latent variables (31). When the square root of AVE for each factor is higher than the correlation with other factors, the scale would be considered to have good divergent validity (32).

Pearson’s correlation analysis and multiple linear regression analysis were applied to evaluate the relationship between subscale scores of the ALS-SF and total scores of the PHQ-9, GAD-7, and Neuroticism subscale. In addition, to explore the differences in affective lability between groups of MDD and BD depression, we also compared subscale scores of the ALS-SF between the two groups using independent samples *t*-tests.

## 3. Results

### 3.1. Demographic and clinical information of participants

The sample of this study was composed of 344 patients, including 221 patients with MDD and 123 patients with BD depression. The mean age of the MDD group and the BD group was 24.59 (SD = 6.091) and 23.12 (SD = 4.015), respectively, and both groups had high proportions of females (MDD = 70.6%, BP = 74.8%). Other demographic and clinical information are provided in Table 1 which showed that there was no significant difference in demographic and clinical characteristics between MDD and BD groups.

**Table 1:** Demographic and clinical characteristics of the MDD and BD groups.

Participant characteristics	MDD (*n* = 221)	BP (*n* = 123)	t/*χ*^2^	*p* value
Age (Mean, SD)	24.59 (4.618)	23.12 (4.015)	1.457	0.147^a^
Gender (*n*, %)			0.372	0.542^b^
Male	65 (29.4%)	31 (25.2%)		
Female	156 (70.6%)	92 (74.8%)		
Residence (*n*, %)			1.505	0.220^b^
Urban	199 (90.0%)	104 (84.5%)		
Rural	22 (10.0%)	19 (15.5%)		
Monthly household income *per capita* (*n*, %)			1.477	0.478^b^
<5,000	89 (40.3%)	45(36.6%)		
5,000–10,000	80(36.2%)	57(46.3%)		
>10,000	52(23.5%)	21(17.1%)		
Education years (mean, SD)	14.00 (2.794)	13.14 (2.201)	1.718	0.088^a^
Current illness duration, months (mean, SD)	9.46(12.286)	9.42(13.575)	−0.017	0.986^a^
Total illness duration, months (mean, SD)	22.56(24.668)	28.21(26.556)	−1.133	0.258^a^
Severity of depression (*n*, %)			3.209	0.002^a^
No (≤4)	0 (0%)	13 (10.6%)		
Mild (5–9)	0 (0%)	9 (7.3%)		
Moderate (10–14)	44 (19.9%)	24 (19.5%)		
Severe (≥15)	177 (80.1%)	77 (62.6%)		
Presence of manic (*n*, %)				
No (<20)	\	12 (9.8%)		
Presence (≥20)	\	111 (90.2%)		

*n*, number of participants; ^a^*p* values obtained by Student’s *t*-test; ^b^*p* values obtained by Pearson’s Chi-square test; Severity of depression measured by total scores of PHQ-9; The presence of manic episodes is assessed by the Young Mania Rating Scale.

### 3.2. Reliability

The Cronbach’s alpha coefficient of the ALS-SF scale was 0.861, and the CR of the three factors ranged from 0.820 to 0.887, indicating that the ALS-SF has good reliability (Table 2). We also found that Cronbach’s alpha decreased after deleting any item, which further suggested that the scale has good reliability and that it was not necessary to delete items from the scale.

**Table 2:** Reliability and convergent validity for the Chinese version of ALS-SF (*n* = 344).

Items of the scales	Factor loading	AVE	CR
Anxiety/depression		0.507	0.834
1. I feel just as relaxed … and then dizzy	0.601^**^		
3. I can be feeling OK and then … jittery and nervous	0.761^**^		
5. I feel nervous … and then … very sad and down	0.817^**^		
6. I go from feeling extremely anxious … to … down	0.788^**^		
7. I shift back and forth from … calm to … nervous	0.555^**^		
Anger		0.487	0.820
4. I … control my temper … to not being able to control it	0.588^**^		
8. I feel perfectly calm … and then … makes me furious	0.840^**^		
9. I will be feeling OK but then I … get mad	0.871^**^		
11. I am so mad … and other times… I get so mad	0.501^**^		
14. I’m so mad that my heart starts pounding	0.612^**^		
Depression/elation		0.496	0.887
2. I have very little energy and then … the same	0.653^**^		
10. I can think clearly … then … difficulty concentrating	0.623^**^		
12. I switch … between … energetic and … little energy	0.728^**^		
13. I feel absolutely wonderful … but soon … the same	0.666^**^		
15. I shift … between unproductive and … productive	0.739^**^		
16. I feel extremely energetic … then … little energy	0.746^**^		
17. I have more energy … then … the same …as everyone	0.802^**^		
18. I’m doing everything … slow but then … I’m no more	0.657^**^		

** Indicates *p* < 0.01; AVE, average variance extracted; CR, composite reliability.

### 3.3. Construct validity

The construct validity of the ALS-SF was examined by EFA and CFA. Results of EFA showed that ALS-SF was appropriate for a factor analysis (KMO = 0.852, Bartlett’s test = 1393.477, *p* < 0.001). Three factors were identified in PFA with eigenvalues of 5.512, 3.303, and 1.743, respectively, and the variance explained by the three factors were 25.076, 17.965, and 15.611%, respectively. The factor loading of all items was greater than 0.5, ranging from 0.587 to 0.850. Based on the item contents of the original scale, three factors were defined as anxiety/depression (AD), depression/elation (DE), and Anger. A three-factor, 18-item scale of affective lability was thus composed for Chinese patients with mood disorders (Table 2). CFA was further conducted to examine the model structure. Results of fit indexes suggested that the scale had a satisfactory goodness-of-fit (RMSEA = 0.070, CFI = 0.918, SRMR = 0.075).

### 3.4. Convergent validity and divergent validity

Table 2 shows the convergent validity of the ALS-SF. All scale items were significantly correlated with the latent variables, with factor loadings ranging from 0.501 to 0.871 (*p* < 0.01), indicating a good convergent validity. The square root of the AVE of each factor is greater than the correlation coefficient of other factors, indicating that the scale has good divergent validity (Table 3).

**Table 3:** Divergent validity.

	AD	Anger	DE
AD	0.710		
Anger	0.616^**^	0.698	
DE	0.223^**^	0.280^**^	0.704

** Indicates *p* < 0.01, AD, anxiety/depression; DE, depression/elation.

### 3.5. Correlation between ALS-SF, anxiety, depression, and personality traits

The results of Pearson’s correlation analysis showed that AD, DE, and Anger were positively correlated with GAD-7 and NEURO. Correlations with GAD-7 ranging from 0.405 for AD and 0.419 for Anger, and correlations with the Neuroticism subscale ranging from 0.278 for DE to 0.55 for Anger. In terms of depressive symptoms, AD was positively correlated with PHQ-9 scale scores (*r* = 0.200), and DE was negatively correlated with PHQ-9 (*r* = −0.307). However, Anger had no significant correlation with PHQ-9 (Table 4). The variance inflation factors (VIF) of the three independent variables were 1.496, 1.178, and 1.572, indicating that there was no significant multicollinearity (VIF < 5) among independent variables. Results of multiple linear regression analyses showed that AD was positively correlated with PHQ-9, GAD-7, and NEURO (*β* = 0.309, 0.217, 0.298, *p* < 0.01); DE was positively correlated with GAD-7 and Neuroticism subscale (*β* = 0.217, 0.198, *p* < 0.01) while negatively correlated with PHQ-9 (*β* = −0.412, *p* < 0.01); Anger had a positive correlation with GAD-7 and NEURO (*β* = 0.216, 0.372, *p* < 0.01), while had no significant correlation with PHQ-9 (Table 5).

**Table 4:** Correlation between affective lability, anxiety, depression, and neuroticism.

	AD	DE	Anger	GAD	PHQ	NEURO
AD	1					
DE	0.276^**^	1				
Anger	0.555^**^	0.334^**^	1			
GAD	0.405^**^	0.363^**^	0.419^**^	1		
PHQ	0.200^**^	−0.307^**^	0.051	0.067	1	
NEURO	0.495^**^	0.278^**^	0.558^**^	0.204^**^	0.202^**^	1

^**^Indicates *p* < 0.01; AD, anxiety/depression; DE, depression/elation; PHQ, Patient Health Questionnaire-9 item; GAD, Generalized Anxiety Disorder − 7 item; NEURO, neuroticism trait of the NEO-FFI.

**Table 5:** Multiple linear regression analyses on the effect of AD, Anger, and DE on PHQ, GAD, and NEUROTICISM.

Variables		*β*	*t*	*p*	*R* ^2^	*F*	*p*
PHQ	AD	0.309	4.611	0.000^**^	0.181	20.976	0.000^**^
	DE	−0.412	−6.917	0.000^**^			
	Anger	0.029	0.420	0.675			
GAD	AD	0.217	3.362	0.001^**^	0.249	30.663	0.000^**^
	DE	0.217	3.776	0.000^**^			
	Anger	0.216	3.261	0.001^**^			
NEURO	AD	0.298	4.631	0.000^**^	0.401	44.551	0.000^**^
	DE	0.198	3.519	0.001^**^			
	Anger	0.372	5.705	0.000^**^			

^**^Indicates *p* < 0.01; AD, anxiety/depression; DE, depression/elation; PHQ, Patient Health Questionnaire-9 item; GAD, Generalized Anxiety Disorder − 7 item; NEUROTICISM, neuroticism trait of the NEO-FFI.

### 3.6. Differences in ALS-SF between MDD (*n* = 221) and BD depression (*n* = 86) patients

Independent samples *t*-tests (Table 6) showed that both factor scores of DE and Anger were higher in BD depression than in MDD (*p* < 0.01). No statistical difference was observed in the AD subscale between the two groups.

**Table 6:** Comparison of AD, DE, and Anger between MDD (*n* = 221) and depressive episode of bipolar disorder (*n* = 86).

	Group	Mean	*t*	*p*
AD	MDD	8.710	−0.926	0.355
	BD (depression)	9.090		
DE	MDD	10.656	−5.324	0.000^**^
	BD (depression)	13.976		
Anger	MDD	6.864	−2.623	0.009^**^
	BD (depression)	8.106		

^**^Indicates *p* < 0.01; AD, anxiety/depression; DE, depression/elation; MDD, major depressive disorder; BD (depression), depressive episode of bipolar disorder.

## 4. Discussion

In this study, we examined the reliability and validity of the ALS-SF in patients with mood disorders in China and investigated the relationship between each factor of the ALS-SF and anxiety symptoms, depressive symptoms, and neuroticism. In addition, we also explored differences between patients with MDD and BP depression regarding their scores on AD, DE, and Anger.

Our study suggested that the Chinese version of ALS-SF had good reliability and validity in general, and the structural model of the ALS-SF derived from the EFA was consistent with the three-factor structural model developed by Oliver and Simons (16). Results of CFA found that the three-factor structural model had a satisfactory goodness-of-fit, convergent validity, and divergent validity. Therefore, ALS-SF is a suitable tool for assessing affective lability in Chinese patients with mood disorders.

Results of correlation analyses showed that scores of three factors of ALS-SF were significantly correlated with PHQ-9, GAD-7, and Neuroticism subscale, except for the Anger subscale which had no significant correlation with PHQ-9. Results of multiple linear regression analyses suggested that AD could be a risk factor that increases depressive symptoms and anxiety symptoms; Anger and DE may both be the risk factors that increase and intensify anxiety symptoms. However, the negative correlation between DE and depressive symptoms indicates that DE may be a protective factor in the development of depressive symptoms. Besides, all three factors were significantly correlated with neuroticism. Neuroticism is a relatively stable personality trait (33) with a high genetic risk (34) and is strongly associated with depressive disorders and bipolar disorder (35, 36). A previous study revealed that neuroticism was significantly correlated with the ALS total score and demonstrated a range of correlations with the subscales (37). Another study carried out on perinatal women found a positive correlation between AD and depressive and anxiety symptoms, which is consistent with our findings (38). Besides, in a large sample follow-up cohort study, irritability in patients with MDD was found significantly associated with anxiety symptoms in that 955 (89.5%) out of 1,067 patients with MDD who reported high irritability also reported anxiety symptoms (39). A similar result was also found in patients with BD (40). However, this study also found that DE had no significant correlation with depressive symptoms, which is different from our findings. Such discrepancy may result from the differences in severity of depressive symptoms and the ability to regulate emotions between perinatal women with mood symptoms and patients with mood disorders.

Results of independent samples *t*-tests revealed that patients with BD depression scored significantly higher than patients with MDD in both DE and Anger subscales, while there was no significant difference between the two groups regarding AD subscale scores. A previous study also demonstrated a similar difference in DE and Anger scores between these two groups (41). The difference in DE may occur because the rapid change between depression and elation is one of the clinical characteristics of BD, while patients with MDD mainly exhibit persistent depression without elation. In terms of higher Anger scores in patients with BD, it was found that anger is a core feature in individuals with BD, presenting in various phases of BD including euthymic, manic, and depressive episodes (42–44). A large sample follow-up cohort study found that the severity of anger before the onset of BD is an important risk factor for the transition from unipolar to bipolar depression (42). During depressive episodes, individuals with BD report about twice the level of anger attacks (62%) reported by those with unipolar depression (45). Surprisingly, the AD subscale did not differ significantly between patients with MDD and BP depression, despite emotional shifts in patients with BD being generally more rapid and intense than in patients with MDD. The reason for such a result remains unclear, which may need further examination in future studies.

Compared with the Chinese version of the ALS-SF validated in the Chinese adolescent population which removed three items (item 2, 4, 10) from the original scale due to non-significant associations with factors (21), our study found that the scale structure of the ALS-SF in individuals with mood disorders remained consistent with the original scale. We believe that such differences may result from the different sample population. Affective lability is one of the key features of mood disorder and has a significant correlation with the development of the disorders (2–4). While in adolescent, affective lability is more likely to be associated with personality types, life events, and external environment (21). In addition, the difference in age may have contributed to the difference in the understanding of items to some extent.

Our study has several strengths and limitations that merit consideration. The total clinical sample size was relatively large in our study, which may facilitate the generalization of ALS-SF for Chinese patients with mood disorders. However, there were some limitations in our study as well. Firstly, due to the relatively small sample size of patients with BD, we were unable to make further comparisons to different subtypes and episodes in BD. Secondly, test–retest reliability analysis was not conducted in our study; thus, we are unable to determine its stability and consistency over time. Thirdly, all participants in this study were recruited from a single clinical center by convenient sampling which may affect the representative of our sample. Further studies should improve the experimental design by (1) including a larger sample size of BD patients and balanced BD subtypes to explore the affective lability of different BD subtypes. (2) The study sample should be derived from multiple centers with a more rational sampling approach to improve the generalizability of ALS-SF. Nevertheless, considering that all the indicators have shown good test results, we believe that the Chinese version of the ALS-SF scale can still be replicated for patients with mood disorders in China.

## 5. Conclusion

To summarize, the Chinese version of ALS-SF exhibits good reliability and validity, indicating that this scale could be a practical tool for clinicians to screen for affective lability of Chinese patients with mood disorders. In addition, our study found that scores of DE and Anger subscales are significantly higher in patients with BD depression than in patients with MDD. Since there is a significant symptomatic overlap between BD depression and MDD, assessing affective lability, especially in the DE and Anger subscales, could potentially assist clinicians to distinguish between the two disorders and make more accurate diagnoses.

## Data availability statement

The raw data supporting the conclusions of this article will be made available by the authors, without undue reservation.

## Ethics statement

All procedures contributing to this work comply with the ethical standards of the relevant national and institutional committees on human experimentation and with the Helsinki Declaration. The study protocol was approved by the Ethics Review Committee of Second Xiangya Hospital of Central South University. Informed consent was provided by all the participants’ parents/legal guardians.

## Author contributions

MM, YJ, and YZ conceptualized the topic. MM wrote the first draft of manuscript. MM, YJ, and YZ reviewed and edited the manuscript. MM, YJ, CX, WO, GL, YQ, XZ, and MH were responsible for the data curation. All authors contributed to the article and approved the submitted version.

## Funding

This work was supported by the National Natural Science Foundation of China (82101612 to YJ), the Science and Technology Innovation Program of Hunan Province (2021RC2040 to YJ), the Natural Science Foundation of Hunan Province, China (2022JJ40692 to YJ), STI2030-Major Projects (2021ZDO202001 to YZ), and the Fundamental Research Funds for the Central Universities of Central South University (1053320211441 to MM). The authors would gratefully acknowledge financial support from the above affiliations. The sponsors had no role in the study design, data collection, and analysis, interpretation of the results and writing of the report.

## Conflict of interest

The authors declare that the research was conducted in the absence of any commercial or financial relationships that could be construed as a potential conflict of interest.

## Publisher’s note

All claims expressed in this article are solely those of the authors and do not necessarily represent those of their affiliated organizations, or those of the publisher, the editors and the reviewers. Any product that may be evaluated in this article, or claim that may be made by its manufacturer, is not guaranteed or endorsed by the publisher.
