# Supplementary material for: Psychometric property study of the Affective Lability Scale-short form in Chinese patients with mood disorders
Source: Front Psychiatry. 2023 Apr 4;14:1160791. doi: 10.3389/fpsyt.2023.1160791 (PMC10110953; doi:10.3389/fpsyt.2023.1160791)
Supplement: Supplementary file 1 [file Table_1.DOCX]

Supplemental Material

Affective Lability Scale – Short Form (Chinese version)

指导语：请仔细阅读以下问题，回忆过去2周内这些描述是否符合您的状态，如果该描述明显不符合您，请选择0；如果该描述多数情况下不符合您，请选择1；如果该描述比较符合您请选择2；如果该描述多半符合您，请选择3。

| 0. 非常不符合  1. 不太符合  2. 比较符合  3. 非常符合 |
| --- |
| 1. 有时我觉得自己和其他人一样放松，然后几分钟内我会变得非常紧张，感到头晕目眩。 |
| 2. 有时我感觉精力不足，但很快我的精力就恢复到正常水平了。 |
| 3. 有时前一分钟我感觉不错，但下一分钟我就会感到紧张不安。 |
| 4. 我经常在能很好控制自己的脾气和根本控制不住自己的脾气之间转换。 |
| 5. 很多时候，我感到特别紧张不安，然后突然变得非常悲伤沮丧。 |
| 6. 有时我对某件事的感受从极度焦虑过渡到非常沮丧。 |
| 7. 我的情绪能在完全平静和紧张焦虑之间来回转换。 |
| 8. 有时我前一分钟还感觉非常平静，下一分钟，最微不足道的事情也会让我大发雷霆。 |
| 9. 我经常有这种时候：当前感觉不错，但突然间我会气得要命，恨不得打什么东西。 |
| 10. 有时我前一分钟还能清晰地思考和集中注意力，但下一分钟我就很难集中注意力和清晰思考了。 |
| 11. 有时候我很生气，几乎不能停止大喊大叫，但很快我就不想再喊叫了。 |
| 12. 我在精力充沛和精力不足以至于去哪都很费劲这两种状态之间来回转换。 |
| 13. 有时候我感觉自己非常棒，但很快我就觉得自己和其他人差不多。 |
| 14. 有时我很生气，我的心脏开始怦怦跳和/或我开始发抖，但很快我会放松下来。 |
| 15. 我在非常没有效率和与其他人一样有效率之间来回转换。 |
| 16. 有时我前一分钟还觉得精力旺盛，但下一分钟我就可能精力不济，几乎不能做任何事情。 |
| 17. 有时我的精力比平时更多，比大多数人都要多，但很快我就觉得自己的精力水平和其他人差不多。 |
| 18. 有时我觉得自己做什么事都很慢，但不久我又觉得自己并不比别人慢。 |

*Note*. This study used the Chinese translation of the ALS-SF from Oliver and Simons (1).

1. Oliver MN, Simons JS. The affective lability scales: Development of a short-form measure. Personality and Individual Differences. 2004;37(6):1279-1288.
